# Post-stroke fatigue: a review of development, prevalence, predisposing factors, measurements, and treatments

**DOI:** 10.3389/fneur.2023.1298915

**Published:** 2023-12-21

**Authors:** Wanjie Chen, Tao Jiang, Huahai Huang, Jingting Zeng

**Affiliations:** Department of Neurology, The Third Affiliated Hospital of Southern Medical University, Guangzhou, China

**Keywords:** cerebrovascular accident, infarction, stroke, fatigue, review, rehabilitation

## Abstract

**Background:**

Post-stroke fatigue (PSF) is a ubiquitous and overwhelming symptom for most stroke survivors. However, there are no effective management strategies for PSF, which is partly due to our limited understanding.

**Objective:**

In this paper, we review the development, prevalence, predisposing factors, measurements, and treatments of PSF.

**Results:**

PSF is an independent symptom after stroke, with a prevalence ranging from 42 to 53%, which depends on the selection of measurement tools and stroke characteristics. It is affected by biological, physical, and psychological factors, among which inflammation may play a key role.

**Conclusion:**

Numerous but non-specific evaluation measurement tools limit the management of PSF. In clinical practice, it may be beneficial to identify PSF by combining scales and objective indexes, such as walking tests and electromyographic examinations. There are no evidence-based interventions to improve PSF. However, increasing evidence suggests that transcranial direct-current stimulation and mindfulness-based interventions may become promising treatments. Further studies are urgently needed to better understand the etiology of PSF, thereby providing the basis for developing new measurement tools and targeted treatments.

## 1 Introduction

Fatigue is a multidimensional motor-perceptive, emotional, and cognitive experience (1). It appears to be a ubiquitous symptom experienced by both healthy and ill individuals. Fatigue plays an overwhelming role for those people with neurological disorders (2). Recently, there was extensive research on fatigue in neurological conditions such as multiple sclerosis (3), Parkinson’s disease (4), and postpolio syndrome (5). However, only a few studies have focused on post-stroke fatigue (PSF).

PSF is a common subjective experience characterized by extreme and persistent feelings of fatigue, weakness, or exhaustion after stroke, occurring mentally, physically, or both, which is not alleviated by general rest (6). Despite accounting for approximately 48% of all strokes (7), PSF often receives little attention from medical practitioners. Previous studies have shown that stroke patients struggle to understand why they feel fatigued, which partly due to a lack of information provided by healthcare professionals (8). Unfortunately, PSF is associated with a range of adverse outcomes, such as impeding functional rehabilitation (9–12), decreasing quality of life (13–15), delaying return to paid work (16), impairing cognitive function (11), and increasing mortality (17, 18).

Despite the high prevalence of fatigue and the significant adverse impact of fatigue on stroke survivors, the effective managements are limited due to being ignored by health professionals. In 2012, stroke survivors and healthcare professionals identified the management and prevention of fatigue as one of the top 10 research priorities related to stroke recovery (19). Therefore, it is crucial to have a comprehensive understanding of PSF. This paper aims to summarize the current evidence on PSF and highlight gaps in the existing research, providing guidance for clinical practice and future scientific investigations.

## 2 Methods

We searched the PubMed Database by combining search terms for relevant disease states, including “stroke,” “fatigue,” “exhausted,” “Fatigue after stroke,” and “Post-stroke fatigue” (inception to October 15, 2023). Two review authors independently scrutinized all titles, abstracts, and reference lists, and excluded obviously irrelevant studies. We utilized the CINAHL, Embase, and MEDLINE databases to access the literature that is not available on PubMed. The review critically compares and contrasts the findings from the above articles.

## 3 The development of post-stroke fatigue

In 1983, Leegaard reported that a large proportion of stroke survivors experienced diffuse cerebral symptoms, manifesting with fatigue, failure of concentration, and others (20). Up to 75% of patients complained that fatigue was the most common symptom. The author attributed this phenomenon to inadequate coping with the consequences of the disease, known as psychological stress response syndrome. It has long been challenging to differentiate between fatigue and depression due to overlapping symptoms after a stroke. Fatigue was often considered a symptom or a risk factor for depression. In 1996, Stein et al. demonstrated that more than 76% of stroke patients experienced fatigue for at least 4 weeks after stroke (21). In addition, the authors noted that fatigue was a weaker indicator of depression than non-somatic symptoms. They explained that somatic symptoms following a stroke may indicate an individual’s behavioral disturbance, but the underlying cause was unknown.

In 1999, Ingles et al. revealed that the frequency of fatigue among stroke survivors 3–13 months after the stroke was higher than in the control group, even among subjects without depression (22). This result suggests that the increased fatigue in stroke survivors cannot be solely attributed to depression. A similar study reported that only 38% of patients with fatigue had elevated depression scores at least 1 year after a stroke (23). Robust evidence suggests that fatigue may occur in stroke patients without any signs of depression, indicating that it is an independent symptom after a stroke. However, there is still no widely accepted definition of PSF.

Fortunately, PSF has been receiving more attention in scientific research and clinical practice. In the 2019 update of the Canadian Stroke Best Practice Recommendations (CSBPR) for Mood, Cognition and Fatigue following Stroke (24), PSF is defined as a multidimensional experience that affects motor perception, emotions, and cognition. It is characterized by a feeling of early exhaustion with weariness, lack of energy, and aversion to effort that develops during physical or mental activity and is usually not ameliorated by rest (Table 1).

**Table 1:** The differences between post-stroke fatigue and post-stroke depression.

Condition	Post-stroke fatigue	Post-stroke depression
Epidemiology	PSF affects 42–53% of stroke survivors (7)	Approximately one third of stroke survivors develop PSD at some point after stroke (25).
Clinical manifestations	A feeling of early exhaustion with weariness, lack of energy, and aversion to effort that develops during physical or mental activity; be unable to relieve through rest (24)	Non-somatic symptoms such as guilt, depressed mood, hopelessness, or worthlessness, which are more likely to suggest PSD (26)
Diagnostic and screening tools	Various but non-specific tools are used, such as Fatigue Severity Scale, Fatigue Impact Scale, and Multidimensional Fatigue Symptom Inventory. The former is the most commonly used scale (7) The Neurological Fatigue Index-Stroke is the stroke-specific scale suitable for screening for PSF (27)	Various but non-specific tools like Patient Health Questionnaire-9 and Hamilton’s Depression Rating Scale are used (25, 28) Most studies use the following diagnostic category to ascertain depression: 1.depressive disorder, depressive symptoms, or psychological distress, as defined by scores above a cut point for abnormality on a standard scale. 2.major depression, or minor depression (or dysthymia) according to the Diagnostic and Statistical Manual of Mental Disorders (DSM-5) (26)
Treatments	Using serotonin reuptake inhibitors (SSRIs) observed no obvious efficacy in two randomized control trials (29, 30)	SSRIs are the primary pharmacological treatment (25)

## 4 Prevalence of post-stroke fatigue

A meta-analysis of 30 studies showed that the prevalence of PSF ranged from 42 to 53% (7). The variation across studies reflects the heterogeneity in measurement tools and stroke characteristics (Table 2).

**Table 2:** Prevalence of post-stroke fatigue.

References	Choi-Kwon et al. (10)	Naess et al. (9)	Van de Port et al. (31)	Winward et al. (32)	Tang et al. (33)	Naess et al. (17)	Duncan et al. (13)	Wu et al. (34)	Kjeverud et al. (35)	Liu et al. (36)	Rahamatali et al. (37)	Zhang et al. (38)
Country, time	Korea, 2005	Norway, 2005	The Netherlands, 2007	United Kingdom, 2009	China, 2010	Norway, 2012	United Kingdom, 2015	China, 2015	Norway, 2021	China, 2020	Belgium, 2020	China, 2023
*N*	220	Stroke: 192 Healthy: 212	223	Stroke: 76 TIA: 73	334	377	136	Stroke: 214 Healthy: 214	93	212	62	230
Stroke types	IS+ICH	IS+TIA	IS+ICH+SAH	IS+TIA+ICH	IS	IS+TIA+ICH	IS+TIA+ICH	IS	IS	IS	All stroke patients	IS (NIHSS<4)
Disease duration	At least 3 mouths	Mean time 6.0 years	At 6, 12, and 36 months	At 6 mouths	At 3 mouths	At least 6 months	At 1, 6, and 12 months	Acute phase	Acute phase	At 6 months	A least 6 months	Within 7 days
Outcome measure	FSS	FSS	FSS	CFS	FSS	FSS	The fatigue case definition	FSS	FSS≥5 FQ Lynch Interview	FSMC	FSS	FSS
Prevalence	57%	Stroke = 51.3% Healthy = 31.6%	6 mouth = 68% 12 mouths = 74% 36mouths = 58%	(1) stroke = 56%; TIA = 29% (2) NIHSS3 = 87%; NIHSS≤3 = 48%	23.4%	42.3%	1 mouth = 33% 6 mouths = 22% 12mouths = 20%	Stroke: 32.2% Healthy: 4.7%	24% (FSS) 62% (FQ) 52% (Lynch interview)	32.1%	71%	31.7%

IS, Ischemic Stroke; ICH, Intracerebral Hemorrhage; FSS, Fatigue Severity Scale; TIA, Transient Ischemic Attack; SAH, Subarachnoid Hemorrhage; CFS, Chalder Fatigue Scale; NIHSS, National Institute of Health stroke scale. FQ, Chalder Fatigue Questionnaire; Lynch Interview, Lynch et al.’s semi-structured clinical interview; FSMC, Fatigue Scale for Motor and Cognitive Functions.

Unless otherwise specified, FSS ≥ 4 is usually defined as fatigue.

One factor that has been claimed to play an important role is different fatigue scales. Anita Kjeverud et al. found significant differences in the reported frequency of fatigue in the same stroke population when using Fatigue Severity Scale (FSS), Fatigue Questionnaire, and Lynch Interview, at 24, 57.62, and 48.52%, respectively (35). A systematic review also found similar discrepancies in patients with subarachnoid hemorrhage (39). The frequency of PSF varied greatly across studies that used different scales: 67 and 71% for the Fatigue Severity Scale (FSS), 36% for the Multidimensional Fatigue Symptom Inventory, and 64 to 90% for a single question (39). Furthermore, using the same scale with different critical values would yield a huge difference. For example, patients with fatigue defined as FSS ≥ 4 or ≥ 5 had a prevalence of 32.1 and 42.3% at 6 months follow-up, respectively (17, 36).

The characteristics of stroke may contribute to the wide range of fatigue frequency. A study revealed that the prevalence of fatigue in stroke patients was 42.3%, while its frequency in transient ischemic attack (TIA), ischemic, and hemorrhagic stroke survivors was 29.6, 45.9, and 19.2%, respectively (17). Notably, the prevalence varies across different stroke stages. Several studies have found that the proportion of fatigue in the acute phase of stroke was around 32% (13, 34, 38), approximately 25% at 3 months after stroke (33, 35, 40), and over 50% at least 6 months (9, 10, 31, 32, 37). The finding suggests a U-shaped pattern, where the prevalence decreases within the first 6 months after stroke and increases since. A systematic review demonstrated that stroke survivors interviewed within the first 6 months had a prevalence of 36%, whereas those assessed after that had a higher proportion of 56% (7), which supported our hypothesis. However, multiple longitudinal studies have shown different patterns of fatigue frequency over time. Additionally, the severity of stroke, as measured by the National Institute of Health Stroke Scale, is also an important factor. Stroke patients with NIHSS scores ≥4 had a higher prevalence of fatigue, reaching 87%, compared to those with lower scores, who had a prevalence of 48% (32).

However, different scales may affect the summary of the current evidence. Therefore, future studies are recommended to unify assessment tools to explore the prevalence at different stages.

## 5 Predisposing factors for post-stroke fatigue

PSF is a multidimensional phenomenon with complex etiology and underlying mechanisms. Some factors might contribute to PSF, including biological, physiological, and psychological factors.

### 5.1 Biological factors

The age and gender of stroke patients are the common and controversial factors. A prospective cohort study reported that older age had a protective effect on PSF at the subacute and chronic stage of stroke (OR 0.94, 95%CI 0.91–0.98 and OR 0.95, 95% CI 0.92–0.98 per year, respectively) (41). However, some studies revealed higher age patients were more likely to experience PSF (17, 36, 42), while others found no age difference (34, 38, 43–45). Similarly, some studies reported no association between gender and PSF (10, 22, 45), while others found female gender was more likely to develop PSF (38, 42, 46). A systematic review of 14 studies showed that the female gender was significantly associated with PSF (OR 1.39, 95%CI 1.14–1.69). This connection did not change in a subgroup analysis of region and follow-up times (47). The disparity could explained by different perceptions, social and family roles, and endocrine differences between genders.

Associations between stroke characteristics and fatigue remain controversial, including severity, location, and subtypes. Winward et al. found that the prevalence of fatigue at the subacute stage of stroke increased with the initial NIHSS (OR 6.96, 95% CI 1.30–49.25) (32). Consistently, a study supported that fatigue was associated with the severity of stroke within 6 months after stroke, while not at the chronic stage of stroke (48). Some studies showed a significant association between stroke locations and fatigue. Tang et al. found that basal ganglia infarction was a significant independent predictor of fatigue after acute stroke (OR 2.084, 95% CI 1.16–3.75) (33). A study reported that basal ganglia, coronal radiation, or internal capsule infarction were independently associated with fatigue 3 months after stroke (43). Similarly, a large-scale study indicated that caudate and putamen infarction were associated with stroke 3 months after stroke, and caudate infarction was a significant independent predictor of PSF (OR 6.4, 95%CI 2.06–20.02). In the study of post-poliomyelitis fatigue and post-viral fatigue syndromes, a brainstem fatigue generator model has been proposed (5). The virus damages the basal ganglia and reticular activating system in areas such as reticular formation and thalamus, resulting in persistent fatigue. Whether post-stroke fatigue shares a similar pathogenesis remains to be studied. Snaphaan et al. found subtentorial infarction was a risk factor for fatigue 2 months after stroke (OR 4.10, 95%CI 1.04–16.12) but not observed at 1.5 years of follow-up (41). Chen et al. also reported that posterior circulation infarction was associated with increased fatigue in the subacute stage but not in the chronic stage (48). No significant association between stroke location and fatigue had not been observed in studies on chronic stroke (10, 42). Likewise, stroke subtypes may be a risk factor for fatigue. Those patients with minor cerebral infarction were significantly more fatigued than those with TIA (17). Su et al. reported that hemorrhagic stroke was significantly associated with acute PSF (46). These results indicate that initial stroke features may be a crucial determinant of early fatigue but are not in later fatigue. However, this conclusion should be treated with caution due to different location classifications. One study found that different classifications of stroke locations, such as supratentorial and infratentorial infarcts, anterior and posterior circulation infarcts, or specific sites, were not associated with fatigue at an average of 15 months after stroke (10). This result cannot be directly applied to patients with acute stroke.

High sensitivity C-reactive protein (hs-CRP) has been identified as a reliable and sensitive inflammatory biomarker (36, 49). C-reactive protein (CRP) is less sensitive than hs-CRP. Wu et al. showed that CRP levels were not significantly correlated with fatigue at 1 and 12 months after stroke, but there was a weak but significant correlation with fatigue at 6 months (*r* = 0.27, 95% CI 0.03–0.48) (50). Liu et al. reported a positive correlation between plasma hs-CRP levels in the acute phase of stroke and fatigue score at 6 months (*r* = 0.369), while hs-CRP was associated with an increased risk of PSF (adjusted OR 3.435, 95% CI 2.22–5.31) (36). Several studies found that hs-CRP or CRP was positively correlated with PSF (49, 51). Thus, hs-CRP levels appear to be a biomarker for the risk of PSF, although the underlying mechanism was still unknown. Nonetheless, previous studies have shown an association between inflammation and other pathological fatigue, such as chronic fatigue syndrome and cancer (52, 53). Thus, immune-inflammatory alterations presumably are involved in the pathogenesis of fatigue. Consistently, a study reported that polymorphism in genes that affect immune response may induce fatigue (54). As is well known, stroke causes an inflammatory cascade in the brain, leading to the activation of inflammatory cells, production of inflammatory cytokines, and changes in surrounding inflammatory cytokine levels (55). How the inflammatory response of stroke causes fatigue has not been fully elucidated. Increasing evidence indicated that elevated pro-inflammatory molecules probably affect the neural and endocrine systems, thus affecting neurotransmitters such as dopamine and serotonin (56, 57). Dopamine reward neurons localized in the ventral midbrain project into the ventral striatum (58), while serotonin fibers from brainstem raphe nuclei project to the basal ganglia (59). The results suggest that fatigue could be attributed to secondary neurotransmitter imbalances resulting from damage in particular locations.

Remarkably, electromyography and electroencephalography may become reliable biomarkers of PSF. We will discuss them in the section on the measurement of PSF.

### 5.2 Physical factors

Current research suggests that medical comorbidities have a prominent impact on later fatigue. A prospective study found that hypertension was associated with lower fatigue scores in chronic stroke but not in acute stroke (48). Several studies have also found a cross-sectional association between PSF and hypertension in the chronic stage of stroke (48, 60, 61) but have not been in acute stroke (13, 34, 38). Diabetes mellitus has also been found to contribute to later fatigue (60, 61) but has not been in the acute stage of stroke (13, 17, 34, 38). The link between PSF and antidepressants or statins was reported (8, 15, 44). Only one study has reported that anticonvulsant drugs and beta-blockers were significant predictors of greater fatigue in patients with chronic stroke (48). It is difficult to draw any conclusion about the relationship between medical history or medication history and PSF. It is difficult to determine whether fatigue is a comorbidity or a drug-related adverse effect.

Pre-stroke fatigue is defined as fatigue lasting at least 3 months before stroke. Wang et al. revealed that the presence of pre-stroke fatigue (OR 4.89, 95%CI 2.13–11.21) was significantly related to fatigue within 2 weeks of stroke after the multivariate adjustment (62). A cross-sectional study reported that pre-stroke fatigue report was associated with higher fatigue scores in the acute phase after stroke (*r* = 0.39) (12). In addition, Chen et al. reported a correlation between pre-stroke fatigue and PSF (*r* = 0.38) (15). Another study also found that pre-stroke fatigue was independently associated with fatigue after 3 months of stroke (OR 33.46, 95%CI 12.25–91.36) (10). They also found that patients with pre-stroke fatigue were more likely to coexist with comorbidities. Thus, it may be related to the long-term physical conditions. However, these conclusions should be treated with caution because of memory bias.

Post-stroke pain has been listed as a contributor to fatigue. Galligan et al. showed that PSF was significantly associated with higher levels of pain in subacute and chronic stroke (*r* = 0.35) (44). Similarly, self-reported fatigue was highly correlated to pain in the chronic stage of stroke (*r* = 0.39) (18). Some studies also suggested that pain was one of the significant clinical variables affecting PSF (17, 44, 63). However, no correlation was found between fatigue scores and pain after 1 year of stroke (45, 60). Post-stroke pain and depression often coexist, but it is difficult to answer which condition dominates in stroke patients. Notable, the assessment of post-stroke pain usually relies on a single question in most studies.

Sleep disturbances, with a prevalence of 78% of stroke survivors (63), have been proposed to aggravate PSF. A cross-sectional study found significant correlations between sleep quality scores and fatigue levels after 1 year of stroke (*r* = 0.31) (45). Rahamatali et al. reported a moderate correlation between fatigue and sleep disturbances in the chronic stage of stroke (*r* = 0.51) (37). Another study found daytime sleepiness was significantly associated with fatigue after 1 and 6 months of stroke (*r* = 0.4, *r* = 0.41, respectively) (13). However, most studies assessed sleep disorders through self-reported scales. Further studies are required to provide more objective data, such as polysomnography, to elucidate the association and causal direction between sleep disturbances and PSF.

### 5.3 Psychological factors

Psychological distress is a broad concept that includes depression and anxiety. One-third of stroke survivors experience fatigue and depressive symptoms (8, 64). The score of the geriatric depression scale was a significant independent predictor of fatigue within 7 days of stroke (OR 1.37, 95%CI 1.25–1.52) (33). Similarly, fatigue was significantly related to depressive symptoms at the acute stage of stroke (OR = 2.39, 95%CI 1.02–5.58) (62). Zhang et al. found that depression has a significant negative impact on fatigue in acute stroke (OR 1.58, 95%CI 1.01–1.98) (38). Mutai et al. reported that fatigue after acute stroke significantly correlated with all multidimensional fatigue inventory dimensions, including general fatigue, physical fatigue, reduced activity, reduced motivation, and mental fatigue (*r* = 0.21, 0.28, 0.40, 0.44, 0.46, respectively). The results indicated that depression had a stronger correlation with reduced motivation, reduced activity, and mental fatigue, which might represent mental components of PSF (65). Several studies also reported a correlation between fatigue and depression at the subacute and chronic stage of stroke. A correlation between Hamilton Depression Rating Scale scores and fatigue scores after 3 months of stroke has been observed (*r* = 0.53) (15). Additionally, self-reported fatigue was highly correlated to depression after 2 years of stroke (*r* = 0.51) (18). Choi-Kwon et al. found that the presence of depression was independently associated with fatigue at an average of 15 months of stroke (OR 2.67, 95%CI 1.04–6.85). Notably, the author also found that the correlation between PSF and PSD disappeared after excluding patients with pre-stroke fatigue. The result suggests that PSD may have some connection with pre-stroke fatigue (10). In a prospective study, depressive symptoms were significantly associated with increased fatigue at 2 months and 1.5 years of stroke (OR 1.36, 95% CI 1.17–1.57; OR 1.30, 95%CI 1.14–1.49, respectively) (41). The connection between PSF and PSD suggests a potential shared mechanism. Fatigue and depression are common symptoms of cytokine-induced disease behavior (66), which serves as a protective regulatory mechanism after infection or tissue damage. This behavior promotes rest and recovery in the short term (67). In the pathogenesis model for clinical depression, pro-inflammatory cytokine signaling in the brain can lead to changes in neuronal function, resulting in sickness behavior, such as reduced appetite and fatigue. However, when risk factors for emotional disorders are involved, the impact can be exacerbated in patients with stronger inflammatory responses or higher brain sensitivity to immune-mediated events (66). PSF and depression may have overlapping pathways, but treatments differ due to varying risk factors. A prospective study with antidepressants in fatigue patients with depressive symptoms would be warranted.

Significant relationships between fatigue and general anxiety (*r* = 0.37), health-related anxiety (*r* = 0.31), and stroke-specific anxiety (*r* = 0.37) have been reported in subacute and chronic stages of stroke (44). A longitudinal cohort study by Duncan et al. illustrated that more fatigue was highly correlated with greater anxiety measured by hospital anxiety and depression score at 1, 6, and 12 months after stroke (*r* = 0.50, 0.52, 0.59, respectively) (13). They also reported that more anxiety at 1 month independently predicted higher fatigue scores at 6 and 12 months. Glader et al. found fatigue was significantly correlated with feelings of anxiety (*r* = 0.42) after excluding the patients with depression (18). Similarly, excluding patients with self-reported depression, a study found that patients experienced more fatigue in those who had signs of anxiety (OR 4.40) (68). In addition, anxiety was significantly correlated with general fatigue and physical fatigue within 2 weeks of stroke (*r* = 0.47, 0.32, respectively), while not with reduced activity, reduced motivation, and mental fatigue measured by multidimensional fatigue inventory (65). It seems that anxiety is more related to the physical components of fatigue, while depression tends to be more associated with its mental components. The long-term effects of anxiety and depression may influence early and later fatigue among stroke individuals. However, the question remains as to whether psychological factors are the cause or consequence of fatigue.

In summary, various factors have been shown to influence fatigue after stroke. Stroke features appear to be more related to early fatigue, and medical comorbidities contribute to later fatigue, while psychological factors play a prominent role in persistent fatigue. However, complex interactions between different factors may be a crucial reason for inconsistent conclusions in studies. Managing these modified factors may decrease the frequency and severity of fatigue and improve the detrimental effects for stroke survivors.

## 6 Measurements of post-stroke fatigue

Fatigue can be categorized as perceived fatigue and fatigability. The former refers to a subjective feeling of energy depletion that disrupts daily activities and is typically assessed by self-report questionnaires. The latter refers to a decline in an individual’s physical performance over time and is measured through objective assessments (37). Several objective measurements with potential applications are currently under study.

### 6.1 Subjective measurements

There are various scales with limited specificity applied to PSF. A systematic review included 48 studies to summarize the prevalence of PSF. The results indicate that FSS is the most frequently used tool, followed by the Multidimensional Fatigue Inventory, Checklist Individual Strength, Fatigue Assessment Scale, Chalder Fatigue Scale, and then Emotional State Questionnaire, Fatigue Impact Scale, Mental Fatigue Scale, Profile of Mood States, and the 36-Item Short Form Survey (7). The Neurological Fatigue Index-Stroke (NFI-stroke) is the only stroke-specific scale with good psychometric properties (27). However, it is more suitable for screening the presence of PSF rather than the severity (69) (Table 3).

**Table 3:** Summary of the scales.

Scale		FSS (70)	MFI (71)	CIS (72)	FAS (73)	CFS (74)	EST-Q (75)	FIS (76)	MFS (77)	POMS (78)	SF-36 (79)	NFI-Stroke (27)
Developed by		Krupp et al. (70)	Smet et al. (71)	Vercoulen et al. (72)	Michielsen et al. (73)	Chalder (74)	Aluoja et al. (75)	Fisk et al. (76)	Bentall et al. (77)	McNair et al. (78)	Ware et al. (79)	Mills et al. (27)
Dimension		The impact of fatigue	General fatigue, physical fatigue, mental fatigue, reduced motivation and reduced activity	Phenomenology and severity of fatigue	Physical and mental fatigue	Physical and mental fatigue	—	Cognitive, physical and psychosocial fatigue	Phenomenology and severity of fatigue	Phenomenology and severity of fatigue	Physical and mental fatigue	Physical and cognitive fatigue
Items (no.)		9	20	8	10	14	33	21	9	6	36	12
Response format		7-point Likert	5-point Likert	7-point Likert	5-point Likert	4-point Likert	5-point Likert	5-point Likert	5-point Likert	5-point Likert	6-point Likert	4-point Likert
Psychometric property in stroke
	Country	Switzerland (80)	NO	NO	China (81)	Iran (82)	NO	Turkey (83)	NO	The United Kingdom (84)	Australia (85)	China (69)
	Reliability (Cronbach’sα)	0.96	NO	NO	0.71–0.82	0.96	NO	0.946	NO	0.88–0.89	>0.7 (except vitality subscale)	0.69–0.88	Validity	NO	NO	NO	Content validity =0.94	Convergent validity = 0.60–0.87	NO	Construct validity =0.82, 0.73, 0.63(cognitive, physical, and psychosocial dimensions)Criterion validity = 0.73	NO	Convergent validity = 0.59, 0.75	YES	Content validity = 0.95 Construct validity = 0.91

FSS, Fatigue Severity Scale; MFI, Multidimensional Fatigue Inventory; CIS, Checklist Individual Strength; FAS, Fatigue Assessment Scale; CFS, Chalder Fatigue Scale; EST-Q, Emotional State Questionnaire; FIS, Fatigue Impact Scale, MFS, Mental Fatigue Scale; POMS, Profile of Mood States; SF-36, the 36-Item Short Form Survey. NFI-Stroke: Neurological Fatigue Index-Stroke.

The effort to improve our understanding of PSF may be hampered by the fact that most fatigue measurements are designed for non-stroke populations and rely on retrospective descriptions by subjects. For instance, the most frequently used scale for PSF (7), the FSS, was originally developed to assess fatigue related to systemic lupus erythematosus and multiple sclerosis (70). It is important to highlight the flaws of current evaluation scales for PSF.

There are several problems with using scales to assess fatigue, such as recall bias and the inability to capture the variability of fatigue. A theory of “the so-called memory-experience gap” suggests that the questionnaires that rely on episodic memory may overestimate actual symptom experience (86). For instance, Lenaert et al. recruited 30 stroke patients to complete 10 questionnaires every day for six consecutive days, and all individuals rated their fatigue retrospectively by FSS on the last day (87). The results suggested that retrospective measurement did not fully capture the authentic experience of PSF, and the same scale score manifested in patients with different daily fatigue modes. Additionally, dichotomizing fatigue patients by a cut-off score, as most researchers often do, is inconsistent with the subjective perception of fatigue.

The current scale covers different dimensions, including fatigue phenomenology, severity, interference with daily life, and the impact on physical, cognitive, and mental functions. However, there are some primary concerns regarding scale content. A recent review used a mixed-methods approach to evaluate content validity in fatigue patient reported outcome measures (PROMs) (88). The review has found that current fatigue PROMs fail to provide a complete dimension of PSF. For example, most items in FSS measure the severity but ignore its characteristics and diurnal variations. Moreover, there is a significant lack of overlap among different PROMs, such as the FSS, which makes it difficult to replicate and generalize findings across studies. Additionally, it is challenging to distinguish whether some items are limited by fatigue or stroke. For example, some items describe the impact of fatigue on functional status and participation in daily life without considering that these dimensions may be directly limited by stroke.

The main reason for the current dilemma is the lack of consensus on what aspects need to be evaluated when diagnosing fatigue in stroke survivors. The diverse and inconsistent fatigue scales have hindered the development of the evidence base for many years. Therefore, there is an urgent need to develop more specific and objective tools for PSF.

### 6.2 Objective measurements

#### 6.2.1 6-min walk testing

The correlation between fatigability and perceived fatigue has been demonstrated in elderly adults (89, 90). A significant relationship has been found between neuromuscular fatigue and perceived fatigue in studies on multiple sclerosis (91). However, there is limited evidence regarding the correlation between these two parameters in stroke subjects. Rahamatali et al. evaluated perceived fatigue and fatigability in patients with chronic stroke by utilizing FSS and the 6-min walk test (6MWT), respectively (37). They defined fatigability as the percent change in meters walked from the first to the last minute of 6MWT. No relationship between fatigability and FSS was reported. However, it is worth noting that the participants only had mild neurological impairments, which may not sufficiently induce fatigability. Further research is needed to determine whether fatigability and perceived fatigue are the same constructs in stroke patients with more severe damage.

#### 6.2.2 Electromyography

Electromyography (EMG), which captures muscle electrical activity sampled from the skin surface and different muscles, may be a promising technology to assess muscle fatigue in stroke survivors. Previous studies have utilized EMG to identify neuromuscular fatigue, including central and peripheral fatigue. The former refers to a progressive contraction-induced reduction in the ability to activate a skeletal muscle voluntarily at the central nervous system, and the latter refers to a loss of force caused by the damage of neuromuscular signal transmission or in the contractile apparatus of the muscle fibers (92, 93). Relevant parameters of EMG have been used as biomarkers of muscle fatigue.

Some studies reported different EMG features of fatigue in stroke survivors. The voluntary activation level measured on the paretic side was lower than on the nonparetic side or healthy individuals (94, 95). During a 20% maximum voluntary contraction (submaximal MVC) fatigue protocol, the neural drive to motor units in both paralyzed and non-paralyzed limbs was greater compared to the healthy group. The finding reflects a declined ability to maximally activate paretic muscles in stroke patients with central fatigue. Stroke survivors have to make more efforts to recruit the muscles to a greater extent to maintain the same relative level of output force. Additionally, fractal dimension (FD) is an index that represents progressive motor unit synchronization in EMG analysis (96), offering insights into muscle condition and neuromuscular control. A decrease in FD was observed, which was sensitive primarily to central fatigue, particularly in young and healthy women (97).

The decrease of power frequency, an index of peripheral fatigue, reflects a reduction of conduction velocity along the muscle fiber membrane. Compared with the nonparetic sides and healthy subjects, there was a significantly less shift toward the lower frequencies of the power spectrum in paretic limbs (98). It also found that the paretic side overall MPF was statistically significantly higher than the nonparetic side and the normal group during the full MVC on the whole time course of the EMG. More importantly, for identifying the fatigue associated with neuromuscular transmission failure, the motor unit firing parameters, including firing rate, minimum inter-pulse interval, and maximum oscillation, were more sensitive than the MPF (98).

EMG may be a useful tool to assess neuromuscular fatigue through electrical alterations due to the destruction of pathways from central nervous system structures to the muscle fibers. In the future, the following directions may be worth considering. Firstly, it is interesting to explore the correlation between muscle fatigue displayed by EMG and self-reported fatigue in stroke patients. Secondly, further research is warranted to explore the potential correlation between improvements in muscle fatigue, as measured by EMG, and reductions in subjective fatigue symptoms.

#### 6.2.3 Electroencephalogram

The alterations in electroencephalogram (EEG) activity caused by fatigue have been investigated in many specific conditions, including fatigue induced by the simulated driving task. Most studies reported that α band power increased and β band power decreased obviously after fatigue (99–101). However, a few studies reported an inconsistent phenomenon (102). Some studies used band power ratios to capture the shift of brain activity from fast waves to slow waves, such as (θ + α) / β and α / β. Two studies reported the related index increased with fatigue during simulated driving tasks, including (θ + α) / β, α / β, (θ + α) / (α + β), and θ / β (100, 103), whereas some studies draw the opposite or unclear conclusion (104, 105). To sum up, the absolute band power or relative band power in EEG used to identify fatigue is still equivocal. The feature of EEG induced by fatigue in stroke patients needs further exploration.

## 7 Treatments of post-stroke fatigue

Currently, there are no evidence-based interventions to alleviate PSF. Only a few clinical trials have explored the effectiveness and safety of different treatments, including pharmacological and non-pharmacological ways (Table 4).

**Table 4:** Summary of trials of interventions for post-stroke fatigue.

Author, Country	*N*	Time after stroke	Intervention Control	Outcome measure	Main findings
Brioschi et al. (106), Switzerland	23	12–48 mouths	I: 50 mg/d modafinil at initial, and up to 200 mg/d at 2 months	FAI	Significant difference between brainstem or diencephalic stroke patients and cortical stroke
Poulsen et al. (107), Denmark	41	Within 14 days	I: 400 mg/d modafinil C: placebo	MFI-20	No significant differences between groups
Bivard et al. (108), Australia	36	At least 3 months	I: 200 mg/d modafinil C: placebo	MFI	Significant difference between groups in favor of modafinil
Karaiskos et al. (29), Greece	60	Within 12 months	I: 60–120 mg/d duloxetine C: 20–40 mg/d citalopram or 50–200 mg/d sertraline	FSS	No significant differences between groups
Choi-Kwon et al. (30), South Korea	83	3–28 months	I: 20 mg/d fluoxetine C: placebo	VAS FSS	No significant differences between groups
Wang et al. (109), China	123	1 week	I: 600 IU/d cholecalciferol+usual care C: usual care	FSS	Significant difference between groups in favor of cholecalciferol
Zedlitz et al. (6), The Netherlands	83	At least 4 months	I: COGRAT C: CO	CIS-F	Significant difference between groups in favor of COGRAT
De Doncker et al. (110), United Kingdom	30	At least 3 months	I: tDCS C: sham tDCS	FSS-7 VAS	Significant difference between groups in favor of intervention
Dong et al. (111), China	60	At least 3 months	I: tDCS C: sham tDCS	FSS	Significant difference between groups in favor of intervention
Johansson et al. (112), Sweden	26 (stroke+TBI)	at least 1 year	I: MBSR C: blank control	MFS	Significant difference between groups in favor of intervention (*F* = 8.47, *p* = 0.008)

FAI, Fatigue Assessment Inventory; MFI, the multidimensional fatigue inventory; FSS, the Fatigue Severity Score; VAS, visual analog scale; COGRAT, cognitive therapy with graded activity training; CO, cognitive therapy; CIS-F, Checklist Individual Strength-subscale Fatigue; tDCS, Transcranial direct-current stimulation; TBI, Traumatic Brain Injury; MBSR, Mindfulness-Based Stress Reduction; MFS, Self-Assessment of Mental Fatigue.

### 7.1 Pharmacological interventions

Modafinil is a wakefulness-promoting drug that promotes the monoaminergic pathways to increase the release of serotonin, dopamine, and orexin (113). It was valid in alleviating fatigue in brainstem or diencephalic stroke but not in cortical stroke survivors (106). This selective effect supports the hypothesis that dysfunction of the reticular activation system is involved in fatigue. However, the study only had 23 samples and lacked a control group. Poulsen et al. reported no significant differences between the participants with 400 mg/day modafinil and a placebo (107). A placebo-controlled trial showed a significant decrease in fatigue for patients who received 200 mg/day of modafinil (108). However, the discrepancy may be explained by the differences in stroke stages and drug dosages.

Two randomized control trials found no significant differences in fatigue stroke patients when using serotonin reuptake inhibitors (duloxetine and fluoxetine, respectively) compared to the control group (29, 30). The results support that depression and fatigue are different and independent diseases.

A retrospective study found that vitamin D supplementation could improve fatigue symptoms in fatigue stroke patients with vitamin D deficiency compared to the control group (109). The mechanism by which vitamin D reduces fatigue is still unclear, but neuroprotective mechanisms may play an important role. Calcitriol, with neuroprotective and neurotrophic actions, is the most biologically active metabolite in vitamin D. It increases blood flow to the central nervous system after stroke by regulating inflammation and inhibiting the increased production of reactive oxygen (114). However, it was only a retrospective study and lacked a placebo-control group.

### 7.2 Non-pharmacological interventions

A randomized controlled trial randomly assigned 83 stroke patients to receive two non-pharmacological treatments, namely cognitive therapy (CO) and CO with graded activity training (COGRAT), respectively (6). The findings reported that both treatments had a beneficial effect on reducing fatigue, while COGRAT yielded greater benefits. In addition, it showed a significant beneficial effect for the COGRAT group on physical endurance measured by the 6-min walk test. The benefit of COGRAT in reducing fatigue may be explained by improving physical endurance. However, the generalizability of CO or COGRAT is limited by physical functioning and cognition requirements.

Noninvasive brain stimulation (NIBS) is a valuable treatment for some neurological diseases, such as multiple sclerosis-related fatigue. One of these NIBS techniques is transcranial direct current stimulation (tDCS). tDCS works through low-intensity direct currents with anode and cathode electrodes placed on the scalp and connected to a battery-driven stimulator (115). Traditionally, different modes of tDCS have different effects, with anode stimulation increasing cortical excitability, whereas cathode stimulation has the opposite effect (116). The regulatory effect of cortical excitability occurs in the stimulated brain area but adjacent brain regions (117). Motor cortex excitability is usually related to attention, perception, and muscle motor function (118–120). Stroke survivors who reported high levels of fatigue had low cortical excitability at rest measured by transcranial magnetic stimulation (121). Central fatigue may originate from low activation on the frontal lobe with a reduced sensitivity to stimuli (5). A sham-controlled intervention study found a single session of anodal tDCS targeted the bilaterally over the primary motor cortex (M1) at 2 mA improved fatigue after 1 week of the treatment (110). Another randomized controlled trial explored the effect of tDCS targeted dorsolateral prefrontal cortex (DLPFC) at 1.5 mA with a continuous treatment plan of 6 times a week for 4 consecutive weeks (111). Compared to the sham tDCS group, the tDCS group significantly improved cortical excitability of the affected hemisphere (122). Some studies found that tDCS could improve the excitation ability of neurons in the prefrontal region and regulate neurotransmitters, such as increasing the level of dopamine and serotonin in the brain (5, 123), which may explain why tDCS can effectively alleviate PSF. Studies applying tDCS for alleviating fatigue in neurological conditions usually used anodal stimulation targeted at the left DLPEC (124). The most common stimulation intensity was 1.0–4.0 mA, lasting 15 to 30 min (124). The above studies with different stimulation intensities indicated that tDCS was beneficial for improving fatigue, but it is hard to obtain consistent evidence. Vaseghi et al. found that a tDCS of M1 and DLPFC significantly increased the brain excitability in M1 in healthy participants (125). A systematic review indicated that applying tDCS targeted at the left DLPFC may produce a beneficial effect in reducing multiple sclerosis-related fatigue, whereas M1 stimulation showed no significant effects (126). Liu et al. supported a greater effectiveness of tDCS in the 1.5 mA subgroup compared with the 2 mA subgroup in patients with multiple sclerosis-related fatigue (127). The heterogeneity of stimulation parameters and the population could play a prominent role in these results. There are still many issues with the application of tDCS in reducing PSF. In the future, large-sample studies should explore the effectiveness and safety of tDCS and analyze its optimal treatment strategy.

Of interest, mindfulness-based interventions (MBIs) have recently received increasing attention. The principle of MBIs is to foster greater mindfulness, which is characterized as a psychological state with a complete focus on the present moment and a non-judgmental awareness of our experiences (128). MBIs have gradually formed standard courses for health in the context of clinically oriented practices, mainly including mindfulness-based stress reduction (MBSR) and mindfulness-based cognitive therapy (MBCT) (129). Both of these interventions focus on mindfulness meditation (MM). In scientific studies, mindfulness interventions come in various forms, ranging from 3-month residential mindfulness meditation retreats to brief single guided mindfulness exercises (130). However, the MBSR program with 8 weeks is the most popular mindfulness intervention program, which includes eight 2.5-h classes, a day of retreat, and daily audio guided home exercises (130). The exact mechanism of MBIs is ambiguous, but it is presumed to affect physical health through biological, health behavior, and psychological pathways (131). A study reported that mindfulness meditation (MM) produced different degrees of relaxation by increasing alpha and theta activity, and a positive emotions pattern by increasing left frontal alpha activity (129). Meanwhile, meditative training may foster the ability to regulate mentation spontaneously and attentional processes (132, 133). Recently, clinical trials demonstrated the potential for MIBs to improve depression, anxiety, substance, alcohol abuse, and chronic pain (129, 131, 134, 135). They showed great benefits in reducing pathological fatigue induced by multiple sclerosis and cancer (112). Thus, MBIs may become a promising strategy for nursing patients with numerous physical and mental health issues. However, little is known about its clinical efficacy in patients with PSF. A study found that MBSR statistically significantly improved mental fatigue in stroke or traumatic brain injury patients compared to the control group. The control group subsequently completed the program and also showed a significant decline in fatigue (112). Importantly, a systematic review found no obvious evidence of harm or adverse events reported in TIA and stroke patients who used MBIs (136). MBIs have a series of advantages. For example, individuals can independently practice after an 8-week course without any requirements for rehabilitation services support. It is convenient to implement within the community, requiring fewer resources and low costs. MBIs may be beneficial to improve fatigue induced by stroke, and may not cause any significant harm. Due to the heterogeneity of diseases, MBIs may not be universally applicable to all stroke survivors. For example, some stroke patients who are in the acute stage of stroke or have severe aphasia will be restricted from engaging in mindfulness practice. At the same time, compared to other diseases, stroke patients are confronted with unique challenges, which require more extensive research on the treatment of the mind. In addition, there are still several limitations of the current study. Firstly, it only has a small sample and poor methodological quality. Secondly, there is no evidence of the efficacy-dose relationship and durability of MBIs. Meanwhile, it is unclear about efficacy differences between pharmacological and non-pharmacological methods. In the future, more high-quality randomized controlled trials are required to confirm its effectiveness.

## 8 Discussion

The purpose of this review is to provide an overview of the development of post-stroke fatigue and summarize evidence regarding its prevalence, predisposing factors, measurements, and treatments, offering insights for future research.

Our study found that different studies have a different prevalence of PSF. We speculate it is similar to a U-shaped pattern, which decreases first and then increases after 6 months. However, several longitudinal studies have drawn different conclusions on the incidence trend. For instance, a study showed that the prevalence of fatigue in the acute stage of cerebral infarction was 23.4%, and increased to 29.6% after 3 months (43). Van de Port et al. reported a frequency of 68% at 6 months after stroke, 74% at 1 year after stroke, and 58% at 3 years after stroke (31). One large-scale study showed a downward trend from 3 months to the first year and remained nearly stable (137). This may be explained by the heterogeneity of assessment tools and population. Nevertheless, PSF has a high prevalence at any stage of stroke.

**Figure 1:**
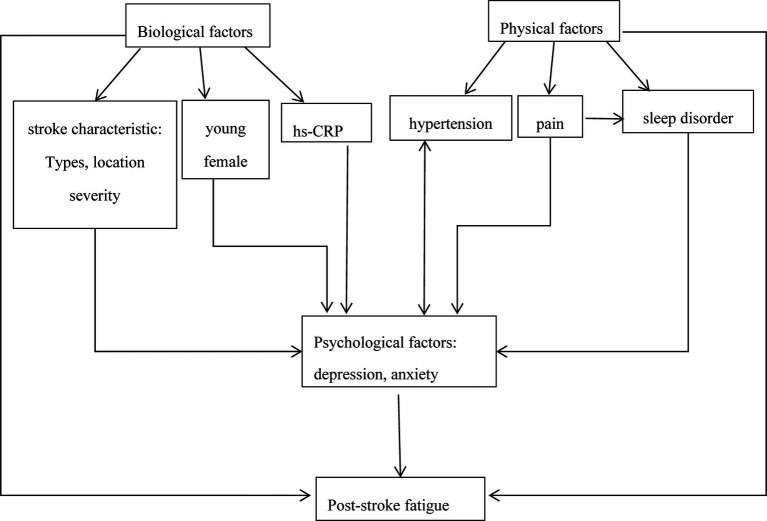
Illustration of the interrelationship between post-stroke fatigue and its factors.

Different studies have varying conclusions on the predisposing factors of PSF, which may be explained by the complexity of fatigue and the interaction between various predisposing factors. By drawing on the literature on fatigue in other diseases and currently available evidence from stroke studies, we propose a model to illustrate the interrelationship between post-stroke fatigue and its predisposing factors (Figure 1). In this model, psychosocial factors play crucial roles in PSF. Previous studies have confirmed that increasing hs-CRP was a potential contributing factor for post-stroke depression or depressive symptoms (138, 139). Additionally, hypertension patients often experience depression, and depression may lead to hypertension conversely through inducing hyperfunction of the sympathetic nervous system (140). In addition, post-stroke pain was associated with sleep disturbances and depression (141). Similarly, many risk factors such as young, female, stroke location, stroke severity, and poor sleep quality were more likely to post-stroke depression (142–144). The interaction of various factors may directly or indirectly induce fatigue, which may confuse current research results. More importantly, it is premature to draw any conclusions about their relationship with PSF due to limited information. Exploring the pathophysiological mechanisms of PSF, notably focusing on the correlation between the pathological pathways of fatigue and depression, may help solve current challenges.

Presently, the diagnosis of PSF mainly relies on various scales, which have many defects, such as non-specific, subjectivity, recall bias, and incomplete dimensions. It is meaningful to develop relatively objective fatigue assessment tools. The existing research evidence for the 6-MWT is insufficient. On the one hand, it may not be sufficient to induce fatigue in those patients with mild stroke, and on the other hand, it is difficult for patients with relatively severe stroke to complete the test. The findings between fatigue and EMG-related parameters are comparatively consistent and promising. However, previous studies reported that those patients without residual neurological deficits, such as transient ischemic attacks and slight mild stroke, still had a higher prevalence of fatigue (17, 32). Thus, it is recommended to explore the differences between electromyographic parameters and subjective fatigue in stroke patients with and without hemiplegia. That may be beneficial to elucidate whether the fatigability and perceived fatigue are the same entity.

There is insufficient evidence in pharmacological therapy for PSF, mainly due to its unclear pathological mechanism. In contrast, non-pharmacological intervention plays a signifiant role. The effectiveness of tDCS on PSF has been preliminarily demonstrated, but large-sample randomized controlled trials are still needed, notably focusing on its target, intensity, and session. For example, Batsikadze et al. found that increasing tDCS intensity might change the direction of excitability changes, and longer or more intensive stimulation did not increase its efficacy (145). A better understanding of its efficacy-dose relationship can provide a specific direction for stroke rehabilitation. In addition, previous studies have shown that tDCS can help improve depression and pain for some neurological system diseases and chronic diseases (115). Similarly, MBIs have been proven to help improve anxiety, depression, and pain (129). It is unclear whether both of them improve fatigue directly or indirectly through improving other predisposing factors. Thus, it is worth exploring the mechanism of reducing fatigue of tDCS and MBIs.

### 8.1 Limitations

A major limitation of this study is that we have primarily researched the Pubmed database, and this may miss some published articles in other databases. However, Pubmed is one of the most authoritative medical literature databases, with sufficient literature coverage and quantity. We have integrated a comprehensive search strategy and supplemented some important literature through other databases. Additionally, we did not include articles written in languages other than English, which may have limited the findings of this study.

## 9 Conclusion

### 9.1 Implication for clinical practice

PSF is a multidimensional and complex phenomenon with an unclear etiology. Clinicians should be aware that PSF, one of the complications of stroke, harms function rehabilitation. They should provide sufficient information about PSF and consider fatigue management in stroke treatment and rehabilitation. The stroke patient objectively shows a decline in performance when performing repeated physical or mental tasks or complains of persistent fatigue at any point during the recovery process. In this context, clinicians should predict the likelihood of PSF and select appropriate instruments to evaluate, among which NFI-stroke may be valid to screen fatigue after stroke. In addition, we should combine other scales and objective instruments, such as walking tests and electromyographic examination results, to make a comprehensive judgment. Considering predisposing factors, it is essential to screen and improve these elements in stroke patients with fatigue. At present, there is a lack of strong research evidence to reduce fatigue after stroke, regardless of pharmacological or non-pharmacological interventions. However, tDCS and MBIs may become promising non-pharmacological treatments for PSF.

### 9.2 Implication for research

In the future, research on post-stroke fatigue should focus on the aspects as follows. First of all, developing fatigue assessment tools for post-stroke fatigue. It is necessary to make clear whether fatigability and perceived fatigue are the same entity and whether they could be evaluated using the same tool. We need more research to explore the characteristics of EMG and EEG in stroke survivors with fatigue. Then, assessing the efficacy of tDCS. Large-scale randomized controlled trials are required to compare the efficacy of tDCS in different populations and intervention plans while considering confounding factors such as depression and pain.

## Author contributions

WC: Conceptualization, Writing – original draft, Writing – review & editing. TJ: Conceptualization, Supervision, Writing – review & editing. HH: Writing – review & editing. JZ: Writing – review & editing.
