# Supplementary material for: Post-stroke fatigue: a review of development, prevalence, predisposing factors, measurements, and treatments
Source: Front Neurol. 2023 Dec 21;14:1298915. doi: 10.3389/fneur.2023.1298915 (PMC10768193; doi:10.3389/fneur.2023.1298915)
Supplement: Supplementary file 1 [file Data_Sheet_1.pdf]

Table 1 The differences between post-stroke fatigue and post-stroke depression.

| Condition | Epidemiology | Clinical | Diagnostic and screening tools | Treatments |
|---|---|---|---|---|
| Post-stroke fatigue | PSF affects 42% to 53% of stroke survivors. | A feeling of early exhaustion with weariness, lack of energy, and aversion to effort that develops during physical or mental activity; be unable to relieve through rest. | Various but non-specific tools are used, such as Fatigue Severity Scale, Fatigue Impact Scale, and Multidimensional Fatigue Symptom Inventory. The former is the most commonly used scale.<br><br>The Neurological Fatigue Index-Stroke is the stroke-specific scale suitable for screening for PSF. | Using serotonin reuptake inhibitors (SSRIs) observed no obvious efficacy in two randomized control trials. |
| Post-stroke depression | Approximately one third of stroke survivors develop PSD at some point after stroke. | Non-somatic symptoms such as guilt, depressed mood, hopelessness, or worthlessness, which are more likely to suggest PSD. | Various but non-specific tools like Patient Health Questionnaire-9 and Hamilton's Depression Rating Scale are used.<br><br>Most studies use the following diagnostic category to ascertain depression: 1.depressive disorder, depressive symptoms, or psychological distress, as defined by scores above a cut point for abnormality on a standard scale. 2.major depression, or minor depression (or dysthymia) according to the Diagnostic and Statistical Manual of Mental Disorders(DSM-5). | SSRIs are the primary pharmacological treatment. |

Table 2 Prevalence of post-stroke fatigue

| References | Country,time | N | Stroke types | Disease duration | Outcome | Prevalence |
|---|---|---|---|---|---|---|
| Choi-Kwon et al. | Korea, 2005 | 220 | IS+ICH | At least 3 mouths | FSS | 57% |
| Naess et al. | Norway, 2005 | Stroke: 192<br>Healthy: 212 | IS+TIA | Mean time 6.0 years | FSS | Stroke=51.3%<br>Healthy=31.6% |
| Van de Port et al. | The Netherlands, 2007 | 223 | IS+ICH+SAH | At 6, 12 and 36 months | FSS | 6 mouth=68%<br>12 mouths=74%<br>36mouths=58% |
| Winward et al. | United Kingdom, 2009 | stroke : 76<br>TIA: 73 | IS+TIA+ICH | At 6 mouths | CFS | (1) stroke=56%; TIA=29%<br>(2) NIHSS3=87%; NIHSS⩽3=48% |
| Tang et al. | China, 2010 | 334 | IS | At 3 mouths | FSS | 23.4% |
| Naess et al. | Norway, 2012 | 377 | IS+TIA+ICH | At least 6 months | FSS | 42.3% |
| Duncan et al. | United Kingdom, 2015 | 136 | IS+TIA+ICH | At 1, 6, and 12 months | The fatigue case definition | 1 mouth=33%<br>6 mouths=22%<br>12mouths=20% |
| Wu et al. | China, 2015 | Stroke: 214<br>Healthy: 214 | IS | Acute phase | FSS | Stroke: 32.2%<br>Healthy: 4.7% |

Table 2 Comtinued

| References | Country,time | N | Stroke types | Disease duration | Outcome measure | Prevalence |
|---|---|---|---|---|---|---|
| Kjeverud et al. | Norway, 2021 | 93 | IS | Acute phase | FSS$\geqslant$5<br>FQ | 24% (FSS)<br>62% (FQ) |
| Liu et al. | China, 2020 | 212 | IS | At 6 months | FSMC | 32.1% |
| Rahamatali et al. | Belgium, 2020 | 62 | All stroke patients | A least 6 months | FSS | 71% |
| Zhang et al. | China, 2023 | 230 | IS<br>(NIHSS$<$4) | Within 7 days | FSS | 31.7% |

Notes: IS=Ischemic Stroke; ICH=Intracerebral Hemorrhage; FSS=Fatigue Severity Scale; TIA=Transient Ischemic Attack; SAH= Subarachnoid Hemorrhage; CFS= Chalder Fatigue Scale; NIHSS=National Institute of Health stroke scale.FQ=Chalder Fatigue Questionnaire; Lynch Interview=Lynch et al.'s semi-structured clinical interview; FSMC=Fatigue Scale for Motor and Cognitive Functions.

Unless otherwise specified, FSS $\geqslant$ 4 is usually defined as fatigue

Table 3 Summary of the scales.

| Scale | Developed by | Dimension | Items (no.) | Response format | Psychometric property in stroke | | |
|---|---|---|---|---|---|---|---|
| | | | | | Country | Reliability (Cronbach's α) | Validity |
| FSS | Krupp et al., 1989 | The impact of fatigue | 9 | 7-point Likert | Switzerland | 0.96 | NO |
| MFI | Smet et al., 1995 | General fatigue, physical fatigue, mental fatigue,reduced motivation and reduced activity | 20 | 5-point Likert | NO | NO | NO |
| CIS | Vercoulen et al., 1994 | Phenomenology and severity of fatigue | 8 | 7-point Likert | NO | NO | NO |
| FAS | Michielsen et al., 2004 | Physical and mental fatigue | 10 | 5-point Likert | China | 0.71-0.82 | Content validity =0.94 |
| CFS | Chalder ., 1993 | Physical and mental fatigue | 14 | 4-point Likert | Iran | 0.96 | Convergent validity = 0.60-0.87 |
| EST-Q | Aluoja et al., 1999 | — | 33 | 5-point Likert | NO | NO | NO |
| FIS | Fisk et al., 1994 | Cognitive, physical and psychosocial fatigue | 21 | 5-point Likert | Turkey | 0.946 | Construct validity =0.82, 0.73, 0.63（cognitive, physical, and psychosocial dimensions）Criterion validity=0.73 |
| MFS | Bentall et al., 1993 | Phenomenology and severity of fatigue | 9 | 5-point Likert | NO | NO | NO |
| POMS | McNair et al., 1964 | Phenomenology and severity of fatigue | 6 | 5-point Likert | The United Kingdom | 0.88-0.89 | Convergent validity= 0.59,0.75 |
| SF-36 | Ware et al., 1994 | Physical and mental fatigue | 36 | 6-point Likert | Australia | ＞0.7 (except vitality subscale) | YES |
| NFI-Stroke | Mills et al., 2012 | Physical and cognitive fatigue | 12 | 4-point Likert | China | 0.69－0.88 | Content validity=0.95 Construct validity= 0.91 |

Notes: FSS: Fatigue Severity Scale; MFI: Multidimensional Fatigue Inventory; CIS: Checklist Individual Strength; FAS: Fatigue Assessment Scale; cfs: Chalder Fatigue Scale; EST-Q: Emotional State Questionnaire; FIS: Fatigue Impact Scale, MFS: Mental Fatigue Scale; POMS: Profile of Mood States; SF-36: the 36-Item Short Form Survey. NFI-Stroke: Neurological Fatigue Index-Stroke.

Table 4 Summary of trials of interventions for post-stroke fatigue

| Author, Country | N | Time after stroke | Intervention Control | Outcome measure | Main findings |
|---|---|---|---|---|---|
| Brioschi et al., Switzerland | 23 | 12-48 mouths | I: 50mg/d modafinil at initial, and up to 200 mg/d at 2 months | FAI | Significant difference between brainstem or diencephalic stroke patients and cortical stroke. |
| Poulsen et al., Denmark | 41 | Within 14 days | I: 400 mg/d modafinil C: placebo | MFI-20 | No significant differences between groups |
| Bivard et al., Australia | 36 | At least 3 months | I: 200 mg/d modafinil C: placebo | MFI | Significant difference between groups in favor of modafinil |
| Karaiskos et al., Greece | 60 | Within 12 months | I: 60-120mg/d duloxetine C: 20-40mg/d citalopram or 50-200mg/d sertraline | FSS | No significant differences between groups |
| Choi-Kwon et al., South Korea | 83 | 3-28 months | I: 20 mg/d fluoxetine C: placebo | VAS FSS | No significant differences between groups |
| Wang et al., China | 123 | 1 week | I: 600 IU/d cholecalciferol+usual care C: usual care | FSS | Significant difference between groups in favor of cholecalciferol |
| Zedlitz et al., The Netherlands | 83 | At least 4 months | I: COGRAT C: CO | CIS-F | Significant difference between groups in favor of COGRAT |
| De Doncker | 30 | At least 3 | I: tDCS | FSS-7 | Significant difference between groups in favor of |

| | | | | | |
|---|---|---|---|---|---|
| et al.,<br><br>United Kingdom | | months | C: sham tDCS | VAS | intervention |
| Dong et al.,<br><br>China | 60 | At least 3 months | I: tDCS<br><br>C: sham tDCS | FSS | Significant difference between groups in favor of intervention |
| Johansson et al., Sweden | 26<br><br>(stroke +TBI) | At least 1 year | I: MBSR<br><br>C: blank control | MFS | Significant difference between groups in favor of intervention<br><br>((F=8.47, P=0.008) |

FAI: Fatigue Assessment Inventory; MFI: the multidimensional fatigue inventory; FSS: the Fatigue Severity Score; VAS: visual analogue scale; COGRAT: cognitive therapy with graded activity training; CO: cognitive therapy; CIS-F: Checklist Individual Strength-subscale Fatigue; tDCS: Transcranial direct-current stimulation; TBI: Traumatic Brain Injury. MBSR: Mindfulness-Based Stress Reduction. MFS: Self-Assessment of Mental Fatigue.

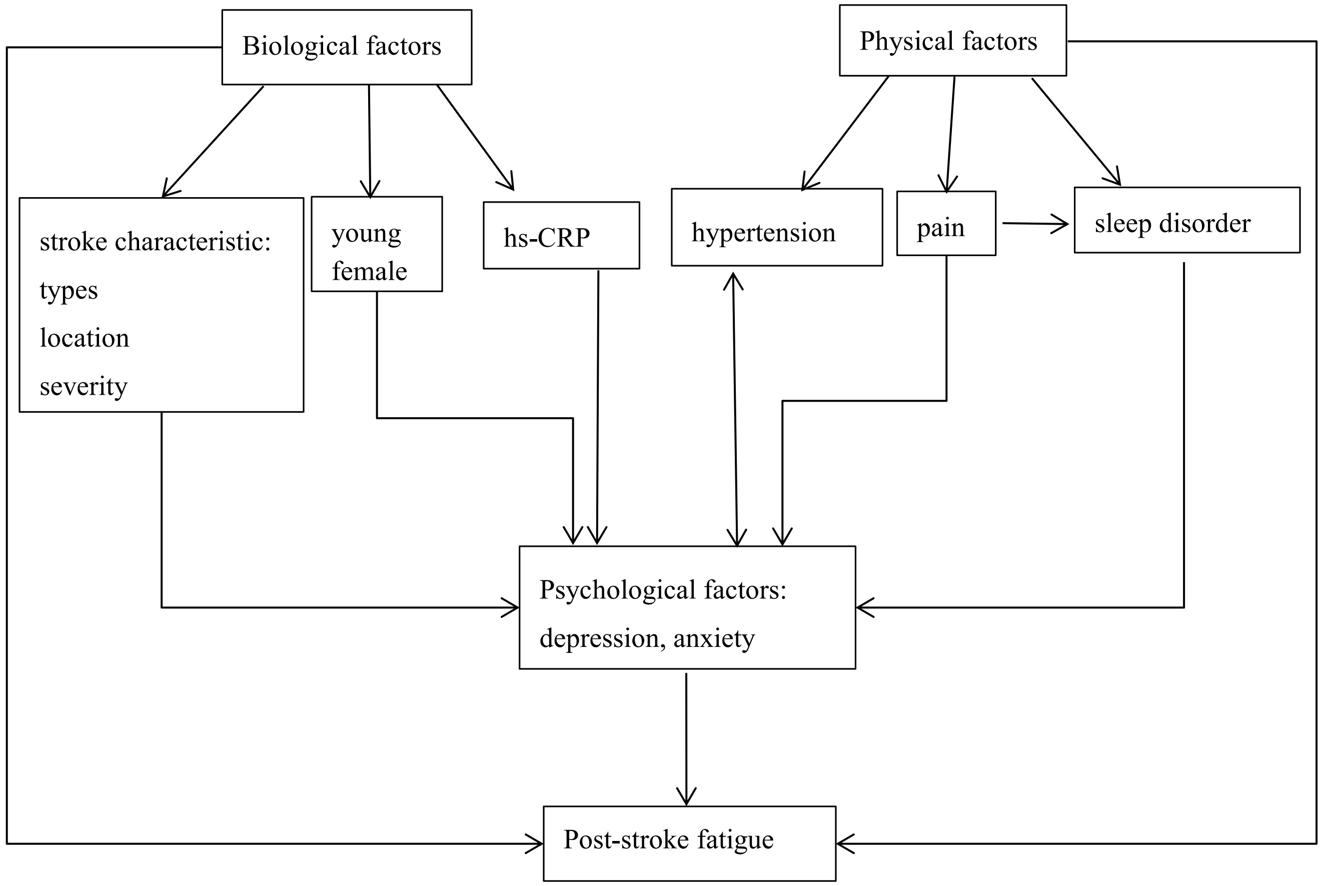


Figure 1 Illustration of the interrelationship between post-stroke fatigue and its factors.
